# Clinical results of autologous protein solution injection for knee osteoarthritis with severe disease grade is inferior to mild or moderate grade

**DOI:** 10.1038/s41598-023-33659-1

**Published:** 2023-04-19

**Authors:** Ayano Kuwasawa, Ken Okazaki, Kuniko Noda, Kotaro Nihei

**Affiliations:** 1 Department of Orthopaedic Surgery, Saitama Cooperative Hospital, 1317 Kizoro, Kawaguchi, Saitama 333-0831 Japan; 2 Department of Orthopaedic Surgery, Tokyo Women’s Medical University, 8-1 Kawadacho, Shinjuku, Tokyo 162-8666 Japan

**Keywords:** Musculoskeletal system, Osteoarthritis, Outcomes research

## Abstract

Autologous protein solution (APS) is made from platelet-rich plasma that extracts high-concentration growth factors and cytokines. Intra-articular APS injection was reported to improve knee osteoarthritis (KOA) pain and function. However, efficacy differences regarding osteoarthritis severity remained unknown. This retrospective study clinically assessed 220 knees with KOA in the Kellgren–Lawrence (KL) grades 2–4 that underwent APS injection using the Knee Injury and Osteoarthritis Outcome Score (KOOS). A telephone survey was performed for patients who dropped out to check symptom changes. The recalculated estimated responder rate included the telephone survey results. The 12-month follow-up was completed with 148 knees (67%), whereas 72 knees dropped out. The follow-up rate was significantly lower in KL4 than KL2 and 3. The KOOS significantly improved in 148 knees, whereas the KOOS was lower in KL4 than in KL2. The responder rate was 55% total, 58% in KL2, 57% in KL3, and 47% in KL4; however, the estimated responder rate, including the telephone survey, was 49% total, 55% in KL2, 54% in KL3, and 36% in KL4. This study showed improved clinical symptoms 1-year after APS injections for KOA, with a lower responder rate in KL4 than in KL2 or KL3.

## Introduction

Knee osteoarthritis (KOA) is a chronic progressive degenerative disease. Conservative treatment is the first choice for relatively mild KOA, whereas surgical treatment, such as knee arthroplasty, is an effective treatment for advanced KOA. Additionally, conservative treatment is needed for advanced KOA that cannot undergo surgical treatment for some reason. Therefore, conservative treatment for KOA is important from a social health perspective because of its high prevalence in an aging society and its important role in disability for mobility.

Conservative treatment for KOA consists mainly of symptomatic treatment with drugs and exercise therapy. The Osteoarthritis Research Society International (OARSI) guidelines^1^ showed that treatments with high consensus levels include oral nonsteroidal anti-inflammatory drugs and exercise therapy, such as walking in water. Meanwhile, intra-articular hyaluronic acid (HA) injections are not recommended. Some other pain control medicines, such as opioids and duloxetine, are recommended in some circumstances, but few conservative treatment options remained recommended for KOA.

Recently, regenerative medicine using one's cells has been attracting attention as a new treatment option for KOA. Various studies focused on platelet-rich plasma (PRP), which concentrates growth factors and anti-inflammatory cytokines that are contained in the blood with expectations for inflammation relief and tissue regeneration^2,3^. Several systematic reviews and meta-analyses of randomized controlled trials (RCTs) suggest that PRP provides more pain or knee function improvements without increasing the risk of adverse events compared with intra-articular injection of HA, analgesics, such as ibuprofen and celecoxib, or placebo treatments in patients with KOA^4–6^.

Autologous protein solution (APS) is made from PRP that extracts high growth factor and anti-inflammatory molecule concentrations through an additional process after preparing PRP, thereby exposing to polyacrylamide beads for dehydration^7^. It is processed from the leucocyte-rich PRP, containing a significantly higher amount of anti-inflammatory molecules, such as interleukin (IL)-1-receptor antagonist, soluble IL-1 receptor II, and soluble tissue necrotic factor (TNF) receptor I, compared with proinflammatory cytokines^7^. The first human clinical safety study of APS by Drumpt et al. reported that a single APS injection in moderate KOA was associated with no serious adverse events and improved pain subscale score of the Western Ontario and McMaster Universities Osteoarthritis Index^8^. Additionally, Kon et al. reported that a single intra-articular APS injection in mild to moderate KOA resulted in significant pain improvement even after 3 years^9^. However, these reports were in cases of mild to moderate KOA with Kellgren–Lawrence (KL) classification grades 2–3, and there were no clinical reports for severe deformities, such as KL4. An RCT with strict inclusion roles revealed that such patients may be difficult to enroll because of ethical considerations. Additionally, information on results with a relatively large number of cases in clinical practice remained inadequate.

APS has been used since 2018, and patients come to our hospital with expectations for a new treatment method, but some patients had severe deformities, such as KL4, which is considered a good candidate for surgery. In such cases, we first recommend surgery, but many patients decline it for various personal reasons and prefer APS therapy. APS therapy was performed for such patients under informed consent. This study aimed to review the APS therapy results in clinical practice and compare the results on the basis of KOA severity in KL grade. This study hypothesized that APS therapy is effective in improving KOA symptoms with a different efficacy in KOA severity.

## Methods

### Patient selection and study design

This retrospective study consists of 12-month clinical outcomes from consecutive 228 knees that are diagnosed with KOA and treated with APS intra-articular injection at our institution from August 2018 to October 2019. Written informed consent was obtained from all the participants.

The main inclusion criteria were ages of 45–85 years, KOA diagnoses with KL grades 2–4 in standing anterior–posterior radiographs, and a history of conventional conservative treatment for at least 3 months (e.g., physical therapy, oral analgesics, and intra-articular injection of HA) that did not provide sufficient benefit. Excluded from the study were patients with rheumatoid arthritis or arthritis secondary to other inflammatory or metabolic diseases, a history of cancer treatment, psychiatric disorders, and other regenerative medicine treatments (PRP, mesenchymal stem cell injections, etc.). In addition, 8 knees in 8 patients who received the intra-articular injection of HA after the APS therapy were also excluded from this study.

KL grade was assessed by an orthopedic surgeon with > 15 years of experience. The classification was repeated for randomly selected 50 knees by the same examiner to evaluate the reliability and reproducibility of the assessment and other examiners to assess the intraobserver and interobserver reproducibility. The concordance rate were 0.91 and 0.92.

APS is prepared using the nSTRIDE APS Kit (Zimmer Biomet). In the first step, 55 mL of blood and 4 mL of anticoagulant citrate dextrose solution A (Citra Labs, Braintree, MA) are injected into the nSTRIDE Cell Separator, and approximately 6 mL of PRP is separated after centrifugation at 3200 rpm for 15 min. The prepared PRP is then transferred to the second step, the nSTRIDE Concentrator, where it is exposed to polyacrylamide beads and filtered by centrifugation at 2000 rpm for 2 min to produce approximately 2–3 mL of APS.

Intra-articular APS injection was performed with a 21G needle within 30 min of creation. After the joint puncture, joint fluid was first aspirated, as much as possible, and then APS was injected. After injection, the patient was instructed to rest at home on the procedure day. The patient is instructed to cool the affected area if knee swelling and pain developed after the injection to relieve the pain, and they were allowed to take one celecoxib tablet as rescue medication if the pain remained strong. For the next day, no activity restrictions were applied, and patients were allowed to gradually resume sports and recreational activities according to their pain tolerance.

No additional conservative treatment, such as medication or joint injections, was given after APS injection in these cases, but any medication given before APS injection was not discontinued. Aspiration of joint effusion was restricted for 1 month after the APS therapy to prevent its effect on APS injection. Aspiration was performed in cases with joint effusion > 1 month after APS injection. The patient was instructed to return for a follow-up examination if joint effusion continues and the patient wants to have the joint fluid aspirated to monitor the joint fluid volume.

### Clinical evaluation

The incidence and duration of acute local inflammatory reaction (AIR), a swelling and burning sensation with pain in the knee joint after APS injection, and the presence of other adverse events were examined to evaluate the safety of APS.

**Figure 1:**
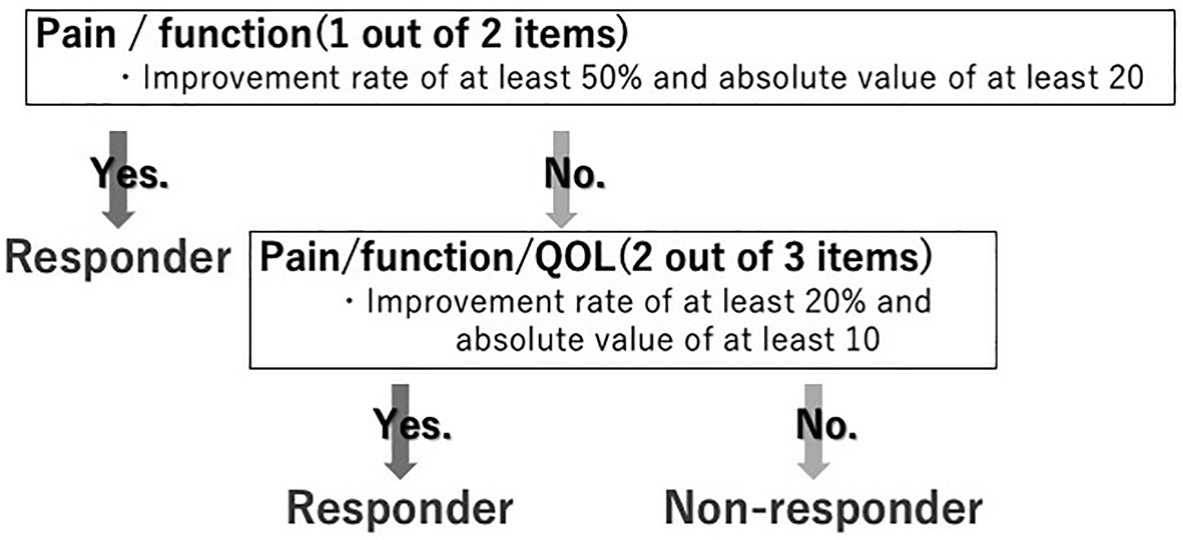
The Outcome Measures in Arthritis Clinical Trials–Osteoarthritis Research Society International (OMERACT–OARSI) criteria^10^.

The Knee Injury and Osteoarthritis Outcome Score (KOOS) was collected before and 1, 3, 6, and 12 months after the APS injection to measure the change from baseline to each time point to evaluate the clinical efficacy of APS. For the patient who did not come to our hospital after 3 months, KOOS was evaluated by mail. The Outcome Measures in Arthritis Clinical Trials–Osteoarthritis Research Society International (OMERACT–OARSI) criteria were used to determine the effect of the APS injection^10^ (Fig. 1). KOOS and efficacy rates were separately compared by KL grade.

Additionally, we examined the details of cases that dropped out from the follow-up within the 12 months. The patients who did not come or renpond to the mail were surveyed via telephone to check for changes in their symptoms. Specifically, patients who were judged as a responder at the last follow-up examination and did not come to the hospital because their condition remained unchanged were considered a responder. Patients who were judged as nonresponders at the last follow-up examination and did not come to the hospital because their condition did not improve were considered nonresponders. Thus, the overall estimated responder rate of the OMERACT–OARSI criteria was recalculated assuming no change in symptoms, and the subsequent efficacy was continued to be determined at the last visit for the clinical evaluation, including the cases who dropped out.

Furthermore, as a worst-case scenario, all the cases that dropped out of the follow-up within the 12 months were considered not effective. The estimated effective rate of the OMERACT–OARSI criteria in the worst-case scenario was also calculated.

### Statistical analysis

Data were described as means and standard deviations (SDs) for continuous variables and as frequency counts and percentages for discrete variables. The Friedman tests were used to detect differences in repeated multiple measures of the KOOS. Dunnett's test was used to compare the KOOS at multiple time points with that at baseline. The Steel–Dwass test was used for the comparison of KOOS among the KL grades. Chi-square tests were used to compare the follow-up rate and efficacy rates in OMERACT–OARSI criteria by KL grades; *p* values of < 0.05 were considered statistically significant. All analyses were performed using the statistical software JMP^®^ ver.15 (SAS Institute Inc., Cary, NC, USA).

### Ethical approval

Following the Law for the Promotion of Regenerative Medicine on Safety of Regenerative Medicine, our submission “Treatment for Osteoarthritis by APS Injection” was accepted by the Health and Welfare Bureau of the Ministry of Health, Labor and Welfare after being reviewed by the Committee for Specific Approval of Regenerative Medicine. Additionally, this study has been submitted to the ethical review board of Saitama Cooperative Hospitall and has been approved as a retrospective study to evaluate the safety and efficacy of the 12-month follow-up results. All data were handled following the Declaration of Helsinki.

## Results

### Follow-up rate

Table 1 shows the details of the patient background. The mean age of the patients was 70.1 years. KL2, KL3, and KL4 included 80 (36%), 74 (34%), and 66 (30%) knees, respectively.

**Table 1:** Patients characteristics.

Gender	Females173 and Males 55
Age	70.1 ± 8.7
Height	157.9 ± 8.4
Weight	60.4 ± 11.8
BMI	24.1 ± 3.2
FTA (°)	179.2 ± 5.8

Mean ± SD. *BMI* body mass index, *FTA* femorotibial angle.

During the 12-month follow-up period, 72 of 220 knees dropped out (follow-up rate: 67%). Nine knees (2 in KL3 and 7 in KL4) underwent total knee arthroplasty during the follow-up period. Table 2 shows the number of knees followed at each time point. Comparing the follow-up rates among the groups, 80% (64/80) in KL2, 73% (54/74) in KL3, and 45% (30/66) in KL4 were observed. The follow-up rate of KL4 was significantly lower than that of KL2 at 6 and 12 months (*p* < 0.01) and that of KL3 at 12 months (*p* < 0.01).

**Table 2:** Number of followed up knees and rates relative to the baseline.

	KL grade 2	KL grade 3	KL grade 4	Total
1 M	80	74	66	220
3 M	72 (90%)	62 (89%)	54 (82%)	192 (87%)
6 M	69 (86%)	54 (73%)	39 (59%)	162 (74%)
12 M	64 (80%)	54 (73%)	30 (45%)	148 (67%)

*KL* Kellgren–Lawrence.

### KOOS

**Figure 2:**
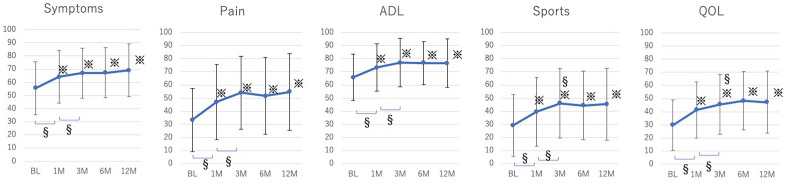
Trend of KOOS in 148 knees followed up until 12 months postprocedure. Mean and SD is indicated. ^※^*p* < 0.05 compared with the baseline in Dunnet's test. ^§^*p* < 0.05 compared between the two time points in paired t-test.

Figure 2 shows the KOOS of 148 knees that completed the follow-up until 12 months. Each item of the KOOS, namely, symptoms, pain, activities of daily living (ADL), sports, and quality of life, was significantly higher at 1, 3, 6, and 12 months after the injection compared with that at the baseline. Additionally, changes in each KOOS item between each time point revealed a significant stepwise improvement from baseline to 1 month and from 1 to 3 months, but no significant change from 3 to 12 months postprocedure.

**Figure 3:**
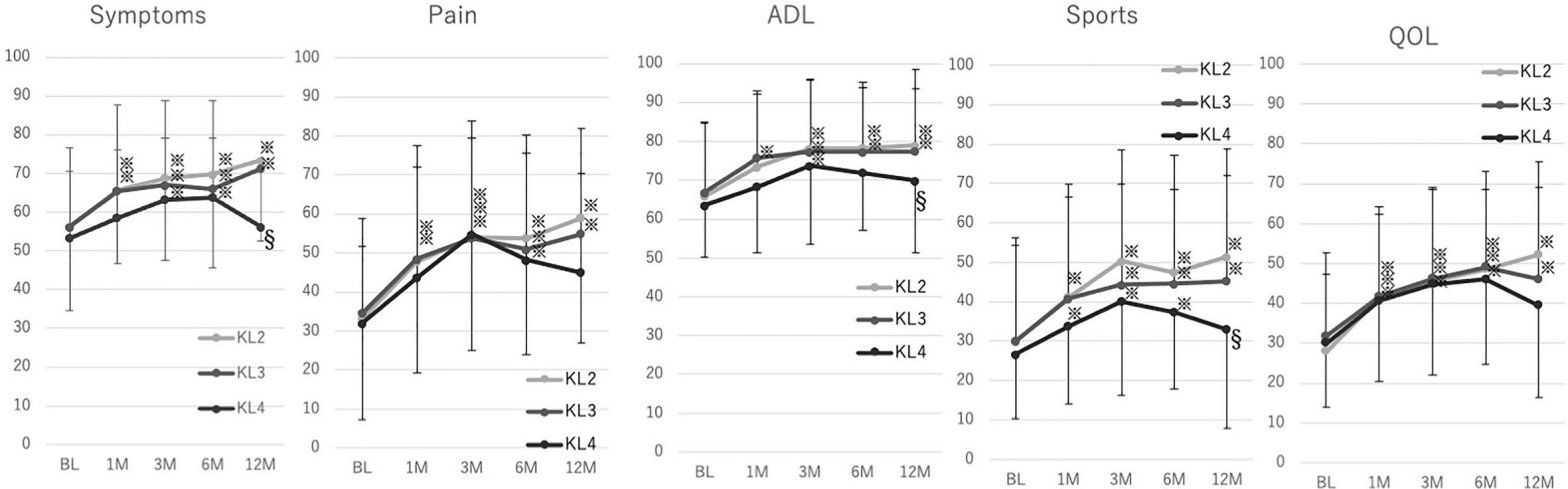
Trends of KOOS in KL grade classifications. Light gray line: KL 2, Gray line: KL 3, Black line: KL 4. Mean and SD is indicated. ^※^*p* < 0.05 compared with the baseline in Dunnet's test. ^§^*p* < 0.05 compared with KL 2 in the Steel–Dwass test.

Figure 3 shows the KOOS of KL groups. KL2 and KL3 significantly improved in a stepwise manner up to 3 months and then the improved status maintained until 12 months. However, KL4 showed similar improvements at 3 and 6 months postprocedure, but the scores at 12 months declined, and the significant improvements disappeared compared with those at the baseline. Comparing the KOOS among the groups at 12 months postprocedure, the KOOS of KL4 was significantly lower than that of KL2 in symptom (*p* < 0.0001), ADL (*p* < 0.05), and sport (*p* < 0.01) subcategories.

### OMERACT–OARSI responder rate

**Figure 4:**
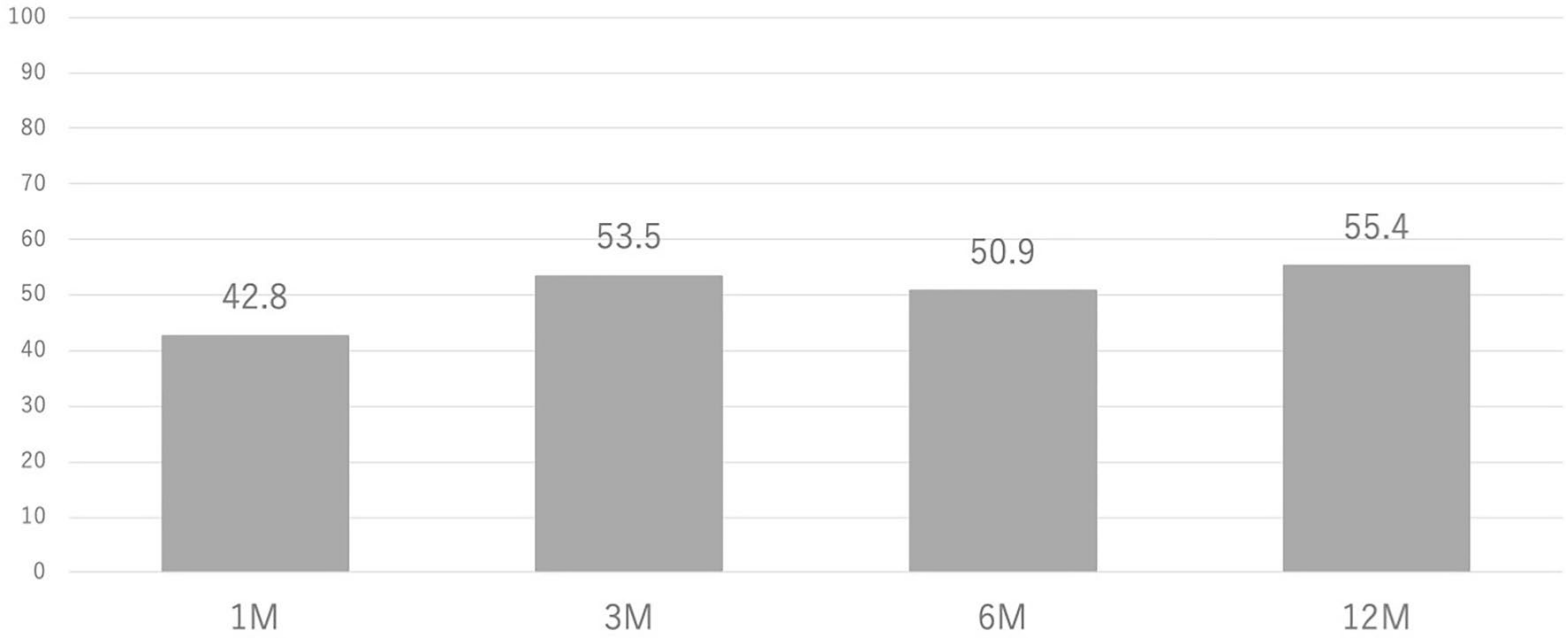
Responder rate in OMETACT–OARSI criteria in the patient followed up at each time point.

The responder rate according to OMERACT–OARSI criteria was calculated. Figure 4 shows the responder rate for the patients followed up at each time point. The overall responder rate was 55.4% (82/148) at 12 months under the 68% follow-up rate. No significant differences were found in the responder rate among the KL grades, but the follow-up rate was significantly low in KL4. The responder rate was recalculated in the following two ways because the difference in follow-up rate has a risk of overestimating or underestimating the results: (1) estimated responder rate under telephone survey for the dropped out cases and (2) estimated responder rate under the worst-case scenario in which all the dropped out cases were considered nonresponders.

The telephone survey for patients who dropped out from the study suggested that 44% (7/16) in KL2, 45% (9/20) in KL3, and 28% (10/36) in KL4 were defined as effective at their last visit and answered as unchanged. The main reason for dropping out from the follow-up included concerns related to coronavirus disease 2019. The rest of the 58 knees (9 in KL2, 11 in KL3, and 26 in KL4) were considered nonresponders. Additionally, 9 out of 58 knees (2 in KL3 and 7 in KL4) underwent total knee arthroplasty during the follow-up period. The nonresponder rate in the dropped out cases was significantly higher in KL4 than in KL2 (*p* < 0.01).

Table 3 shows the summary of the responder rate. The estimated responder rate under the telephone survey were included in the followed up cases. The estimated responder rates at 12 months were 49% (108/220) in total, 55% (44/80) in KL2, 54% (40/74) in KL3, and 37% (24/66) in KL4. There was a significant difference in responder rate among the KL groups (*p* = 0.0468). KL4 tended to be lower than KL2 and KL3. In the worst-case scenario, in which all the dropped out cases were defined as nonresponders, the responder rates were 37% in total, 46% in KL2, 41% in KL3, and 21% in KL4. The responder rate in KL4 was significantly lower than that in KL2 or KL3 (*p* < 0.01).

**Table 3:** Responder rate in the OMERACT–OARSI criteria.

	Actual responder rate	Dropped out (estimated responder)	Estimated responder rate	Responder rate at the worst-case scenario
Total	55% (82/148)	72 (26)	49% (108/220)	37% (82/220)
KL2	58% (37/64)	16 (7)	55% (44/80)	46% (37/80)
KL3	57% (31/54)	20 (9)	54% (40/74)	42% (31/74)
KL4	47% (14/30)	36 (10)	36% (24/66)	21% (14/66)

*KL* Kellgren–Lawrence.

### Adverse effects

Regarding the APS therapy safety, AIR, which caused knee joint swelling and pain within 24 h after APS intra-articular injection, occurred in 62% (137/220) of knees. Patients reported that the AIR duration was within 24 h in 36% (50/137), 1–3 days in 42% (58/137), and 3–7 days in 21% (29/137) of knees. The average duration was 2.8 days. No injection site infections, bleeding, or other adverse events that were considered related to the APS therapy occurred.

## Discussion

The most important findings of this study are that a single injection of APS for mild to moderate KOA can produce significant pain improvement up to 12 months after the injection; however, the effect did not sustain for severe KOA with KL4 and was inferior to that for mild to moderate KOA with more patients dropping out for the 12-month follow-up, although some patients showed symptom improvement for a short period up to 6 months. According to the OMERACT–OARSI criteria, the estimated responder rate at 12 months under the telephone survey for patients who dropped out was 55% in KL2, 54% in KL3, and 36% in KL4. The estimated responder rates in the worst-case scenario, in which all the dropped out cases were defined as nonresponders, were 37% in total, 46% in KL2, 42% in KL3, and 21% in KL4.

Inflammatory reactions have been suggested to play important roles in osteoarthritis (OA) symptoms and pathology. Many studies reported that proinflammatory cytokines and catabolic proteins, such as IL-1β, TNFα, and matrix metalloproteases (MMPs), are involved in cartilage destruction and OA progression^11^. Livshits et al. reported that patients with KOA with high IL-6 levels in the joint fluid have a faster radiographic disease progression^12^. Therefore, the approach of intra-articular administration of high anti-inflammatory cytokine and anabolic factor concentrations can be a potent therapy to protect or prevent OA progression and improve the symptoms of OA. The nSTRIDE APS Kit can extract high concentrations of anti-inflammatory cytokines from autologous blood. APS from patients with KOA has been reported to contain a significantly higher amount of anti-inflammatory molecules, such as IL-1-receptor antagonist and soluble TNF receptor I, compared with proinflammatory cytokines^7^. Other in vitro studies have shown the anti-inflammatory effects of APS in chondrocytes, macrophages, and cartilage matrix^13,14^. Particularly, significant suppression of MMP-13 production was reported when APS was added to chondrocytes with TNFα or IL-1^15^. Additionally, an in vivo study using a rat model of OA after meniscectomy showed a significantly decreased cartilage degeneration score and improved total joint score after APS administration, suggesting its inhibitory effects on degeneration^16^.

Regarding the clinical results of APS, Drumpt et al. conducted a clinical trial for KOA in KL2 and KL3 and revealed that some patients continued to experience symptom improvement for at least 18 months^8^. Kon et al. also reported significant pain improvements after APS injection compared with the placebo injections with saline for up to 12 months^17^, and the effect was observed even after 3 years in patients with KOA with KL2 and KL3^9^. The effect of APS in improving symptoms at 12 months in patients with KL2 or KL3 is suggested to demonstrate better outcomes over that of placebo although our study does not have controls aiming to review the results of clinical practice. The KOOS and responder rate for KL2 and KL3 in our study were similar to those in previous studies, however, those of total patients were relatively lower because of the lower efficacy for KL4. As a predictor of responder, Wu et al. reported with corticosteroid injection showing that the severe pain at the baseline would be its predictor^18^. In our study with APS, however, the worse pain score at the baseline was not a predictor of responders.

Moreover, the efficacy of KOOS peaked at 3–6 months for severe KOA with KL4, and many patients dropped out because of lack of efficacy. Additionally, the estimated response rate of 36% is not much different from the efficacy rate of saline and HA used as controls in previously reported studies^19^. Information regarding the clinical results of APS for severe KOA with KL4 has been inadequate, and some reports suggested that the effect of PRP for KL4 declines within 1 year^20,21^. The current study revealed that APS is unlikely to improve symptoms in patients with severe KOA with KL4. Contrastingly, Weber et al. reported that the OMERACT–OARSI effective rate was 86.1% for TKA 1 year after surgery^22^. Therefore, we consider the obtained data in this study valuable to share the information on APS therapy efficacy based on OA severity and discuss with patients who request this therapy about choosing the optimal therapy for them.

In this study, the frequency of AIR with APS was relatively high compared with the highest rate reported for other PRPs being 29.2%^23^. This may be due to the character of APS, which contains a high number of leucocytes. A previous study suggests a possible catabolic effect of leucocyte-rich PRP due to the release of catabolic or inflammatory molecules from leucocytes in vitro^24^. Another study reported a relatively high incidence of AIR in leucocyte-rich PRP^25^. Meanwhile, a recent in vivo study revealed no increased inflammatory molecules in synovial fluid 1 week after leucocyte-rich PRP injection^26^. Another study showed a little difference in adverse effects between leucocyte-rich and leucocyte-poor PRPs^27^. Therefore, the AIR of the APS therapy and its relationship with leucocytes or other factors need further investigation.

This study has several limitations. First, this retrospective study reviewed the results in clinical practice of APS therapy without controls. Therefore, these results cannot judge the superiority of APS therapy over other therapies, including the placebo. However, the strength of this study is that it compared the results among the severity of OA grades, including KL4, with a relatively large number of cases. Few studies have investigated the clinical results of APS and PRP for severe OA. Second, the follow-up rate for KL4 was lower than that for other groups. A telephone survey was performed to review the symptoms of patients who did not come to our hospital because the lower follow-up rate causes a risk of underestimation or overestimation of the responder rate. However, the telephone survey results might not be accurate.

## Conclusions

Significant KOOS improvements were observed 12 months after the APS therapy for KOA. The responder rate in the OMERACT–OARSI criteria was 55% under the 68% of follow-up rate. The results in patients with KL4 showed lower KOOS and lower follow-up rates than those with KL2 or KL3. The inclusion of the telephone survey for patients who dropped out from the follow-up suggests a lower responder rate in patients with KL4.

## Data Availability

The datasets generated during and/or analysed during the current study are available from the corresponding author on reasonable request.
